# Optimization of the acetic acid method for microfossil extraction from lithified carbonate rocks: Examples from the Jurassic and Miocene limestones of Saudi Arabia

**DOI:** 10.1016/j.mex.2022.101828

**Published:** 2022-08-28

**Authors:** Muhammad Hammad Malik, Septriandi A. Chan, Lamidi O. Babalola, Michael A. Kaminski

**Affiliations:** a Geosciences Department, King Fahd University of Petroleum & Minerals, Dhahran 31261, Saudi Arabia; b Center for Integrative Petroleum Research, King Fahd University of Petroleum & Minerals, Dhahran 31261, Saudi Arabia

**Keywords:** Micropaleontology, Acetic acid disaggregation, Carbonate, Saudi Arabia

## Abstract

An optimization experiment with different acid concentrations was carried out to assess the use of acid to minimum sustainable limits for the extraction of microfossils from indurated limestones. Two different limestone formations of Jurassic and Miocene ages were tested. Different concentrations of acid ranging from 50 to 100% and processing times varying from 2 to 10 h were tested for optimal recoveries. The acid residue recoveries show a similar trend for both formations. The weight percentage of residue with particle size >1 mm decreased as the acid concentration increased, especially in the 50–80% acid concentration range. On the other hand, the weight percentage of the smallest size particles > 0.063 mm increased as acid concentration increased. This means that the higher concentrations of acid dissolve more of the unnecessary large particles while the foraminifera, which comprise the sand fraction size, are left in the residue. Although higher acid concentrations with longer reaction times yielded better recoveries than with less reaction time, we recommended a 60% concentration of acetic acid and a reaction time of 10 h for optimal recovery of micropaleontological samples in Saudi Arabian carbonate rocks. By lowering the recommended concentration, the consumption of acid is reduced without compromising the recovery of microfossils.

• Acetic acid leaching method is applied on two different age limestone samples to extract foraminifera.
• Different concentrations of acetic acid are tried and tested, and consensus is made on an optimum concentration of 60% for a submersion time of 10 h.
• The sample recoveries are optimal while using this concentration for a time of 10 h.

## Graphical abstract

**Figure:**
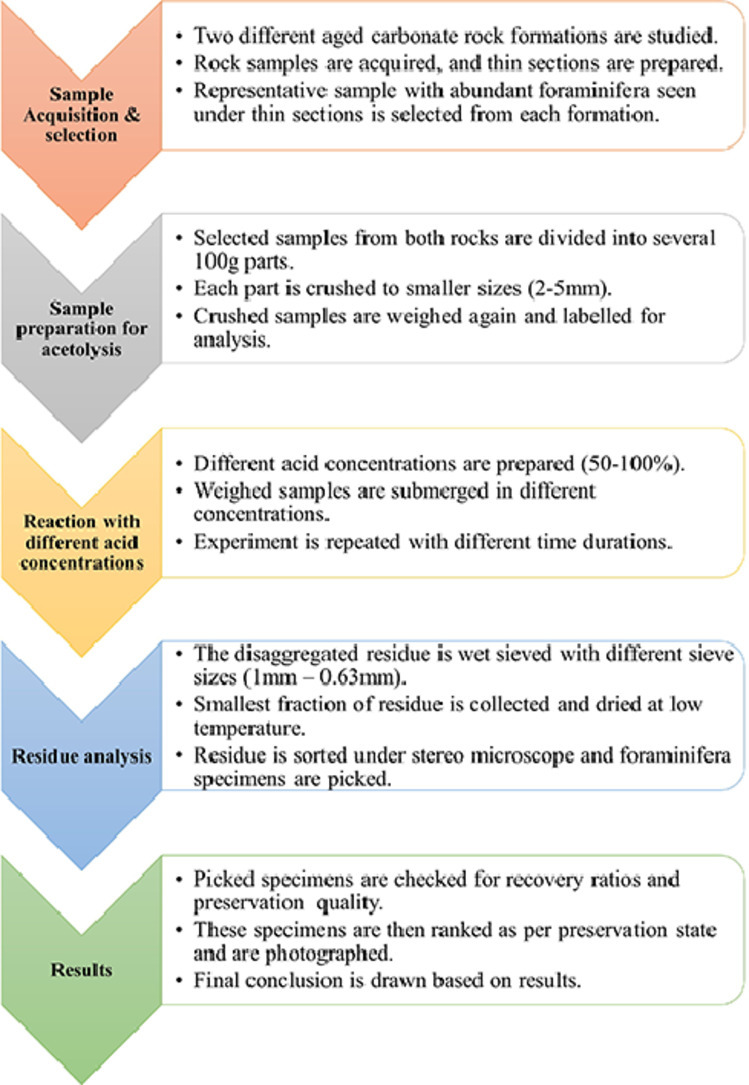

**Specifications table**

**Table:** 

Subject Area:	Geology
More specific subject area:	Micropaleontology
Name of your method:	Acetic acid leaching technique for microfossils for microfossils extraction
Name and reference of original method:	Méthode de dégagement des microfossiles par acétolyse à chaud. Compte Rendu Sommaire des Seances de la Société Géologique de France 1962, 267–268. Bourdon, M., 1962.
Resource availability:	Limestone rocks, Acetic Acid

## Method details

The standard method for microscopic investigation of microfossils in most parts of the Middle East has been through the use of polished thin sections, and the vast majority of recently published studies have used them [1], [2], [3], [4], [5], [6], [7], [8], [9], [10], [11], [12], [13], [14]. Thin sections have been extensively used by the petroleum industry to study microfossils, and foraminifera in particular, from the lithified carbonate reservoirs [15], [16], [17], [18]. Unfortunately, examining foraminifera in thin section has its obvious limitation – as it only allows a two-dimensional view of the specimen. Consequently, taxonomic identification can be problematic, especially for smaller benthic and planktonic foraminifera [19,20]. This is due to the difficulty in identifying and distinguishing the species and even some genera [21]. Polished thin sections can work well for larger foraminifera as well as for rapid biozonal identification in petroleum exploration, but they do not provide enough details for the investigations of species morphological characteristics, such as chamber arrangement, wall structure, and surface ornamentation. It can also be problematic to obtain a large enough dataset to carry out paleoenvironmental studies.

The use of hydrogen peroxide (H_2_O_2_) and sodium carbonate (Na_2_CO_3_) are among the common techniques used for disaggregating marls and marly limestones, and these methods have been applied to many Jurassic and Cretaceous units in the Middle East [22], [23], [24], [25], [26]. These disaggregation techniques, however, are ineffective when applied to strongly lithified limestone. The acetic acid (CH_3_COOH) method therefore is considered as one of the best methods for extracting foraminifera from lithified carbonate rocks without destroying the fossil content [27], [28], [29]. This method has been used by various authors in different parts of the world to extract microfossils from hard, lithified limestone formations, but there appears to be no standardized methodology.

The acetic acid method was first used by Bourdon [30], to extract ostracods from limestone samples. Several researchers [19], [20], [21],^27^,^29^,[31], [32], [33], [34], [35], [36], [37] have successfully used and modified with different concentrations reaction duration (6 h to 40 days), and different sample sizes to extract foraminifera from hard, lithified argillaceous limestones (Table 1).

**Table 1:** Summary of previous studies using acetic acid method for extraction of fossils from lithified carbonate rocks.

Authors	Year	Sample size	Concentration	Reaction Time	Remarks
Bourdon	1962	–	99.5% Acid	–	Early procedure developed and applied to the extractions of ostracods
Notzold	1965		–	30–40 days	Carbonate microfossil separation from hard limestone. Unpublished ms Geology
Stouge et al.	1983	5 cm	10–15% Acid 85–90% H_2_O	1 week	Less concentration of Acid Change of acid 2-3 times No ultrasonic cleaner
Thomas and Murney	1985	3–5 cm	200–250 ml concentrated acetic acid	21 days	Long day of processing the samples there is no information about the percentage of acid Acetic acid mixed with 15–20 g anhydrous copper sulphate
Lethiers and Crasquin-Soleau	1988	2 cm	99.5% Acid	1 day–3 weeks	Modified earlier procedure by Bourdon (1957, 1962). Immersed with acid and place the sample over a hot plate with temperature 60–80 °C, leave for several hours
Wernli and Gorog	1999	–	99.5% Acid	–	500 g sample have been processed in similar way with [34]
Lirer	2000	5 mm	80% Acid 20% H_2_O	4–10 h	No change of acid Used ultrasonic cleaner Marly Limestone and calcilutite only need 4 and 6 h dipped in acetic acid but strong lithified limestone needs 10–15 h.
Holcová	2002	1 cm^3^	5%, 10%, and 30% acetic acid	3–4 weeks	Acid was completely changed every week 10% shows good results because the foraminifera abundant on this concentration.
Reolid	2004	5 mm	80% Acid 20% distilled water	10 h	Followed the disaggregation method from Lirer (2000). Compare with Amine-O Method and Thin section
Patruno et al.	2011	5 mm	80% Acid 20% distilled water	10 h	200 g samples were processed follow the disaggregation method from Lirer (2000).
Rodrigues et al.	2012	0.5 cm	Glacial Acetic acid Hydrochloric Acid Formic Acid Phosphoric Acid Hydrogen Peroxide	–	Evaluated different reaction and reagents conditions in order to determine the best and safest techniques for disaggregation of dolomite rocks for the recovery of ostracods. However, the study can be used to extract other calcareous microfossils.
Hjálmar- sdóttir et al.	2013	–	10% Acid	2 Weeks	1.5–7.65 kg of samples were digested for two weeks.
Coccioni and Silva	2015	5 mm	80% Acid 20% distilled water	10 h	Following the disaggregation method from Lirer (2000). Recovery of Planktonic Foraminifera allows more precise placement of several bioevents and describe species which not recognizable on previous study.

The aim of this study is to optimize the acetic acid method by testing different concentrations of acetic acid on limestone samples from two different economically important formations in Saudi Arabia. The formations are the Middle Jurassic Dhruma Formation, which is exposed near Riyadh and contains three important hydrocarbon reservoirs in the subsurface (Fridah, Sharar and the Lower Fadhili), and the Middle Miocene Dam Formation exposed in the Lidam area of the Eastern province of Saudi Arabia. The equivalents of the Dam Formation in the Arabian Gulf region and Iraq are also important offshore reservoirs.

We tested the acid residue recoveries obtained by reducing the acid percentage from 80% as proposed by Lirer [27] to 50, 60, and 70%, and we compare the results of using different acid concentrations in terms of fossil recovery, test preservation, specimen cleanliness, and assemblage composition. In this study, we also tested stronger concentrations of acid with less reaction time, i.e., five hours for 90% concentration and two hours for 100% concentration. Encouraging results using the acetic method have been produced in recent publications by the authors [24,^38^,39].

## Material and methods

### Rock samples

**Fig. 1:**
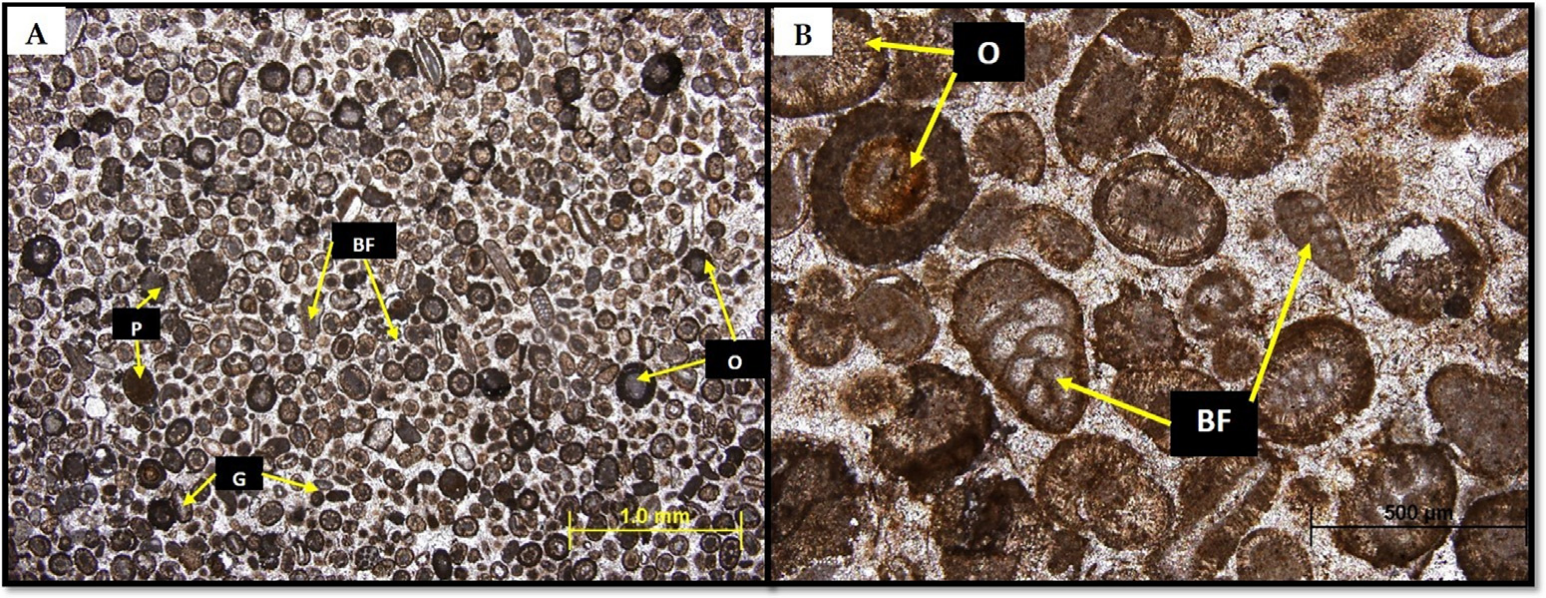
(A) Thin section of Middle Jurassic Dhruma Formation described as oolitic foraminiferal grainstone, mostly composed of oolites with bioclastic fragments, i.e., benthic foraminifera (BF), gastropod (G) etc. Some psoids (P) can also be seen. (B) The closer view of the lithofacies dominated by agglutinated benthic foraminifera (BF) and oolitic grains (O).

Two sets of carbonate samples of different ages and depositional environment were selected for this study. One set of samples is from the Middle Jurassic Dhruma Formation exposed west of Riyadh. The Dhruma Formation is composed of alternating layers of limestone and marls deposited in an offshore carbonate ramp environment [39]. The sample used for the study was taken from a hard, foraminiferal oolitic grainstone unit from the middle part of the formation. The rock is mainly composed of bioclastic skeletal grains including benthic foraminifera, echinoderms, brachiopods, and mollusk fragments. Non-skeletal grains are dominated by ooids with a few peloids. The grains are highly cemented, and few alterations can be seen (Fig. 1).

**Fig. 2:**
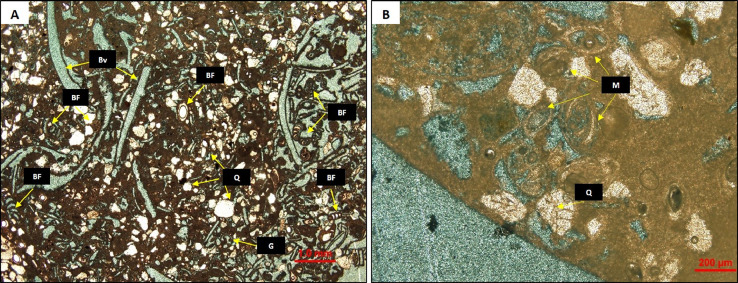
(A) Thin section of Middle Miocene Dam Formation described as skeletal grain dominated packstone lithofacies. It is mostly composed of bioclastic fragments i.e., benthic foraminifera (BF), bivalves (BV) and gastropods (G) and also some siliciclastic quartz grains (Q). (B) closer view of the lithofacies dominated by miliolids (M) and clastic quartz grains (Q).

The second set of samples was collected from the Middle Miocene Dam Formation in the Lidam area. Based on thin section petrography, the sample selected for study is from a skeletal grain-dominated packstone lithofacies. The limestone is grain supported and mostly composed of benthic foraminifera, bivalves, and gastropods, with some quartz grains (Fig. 2), and was deposited in a shallow-water, possibly hypersaline, environment [38].

### Methodology applied

**Fig. 3:**
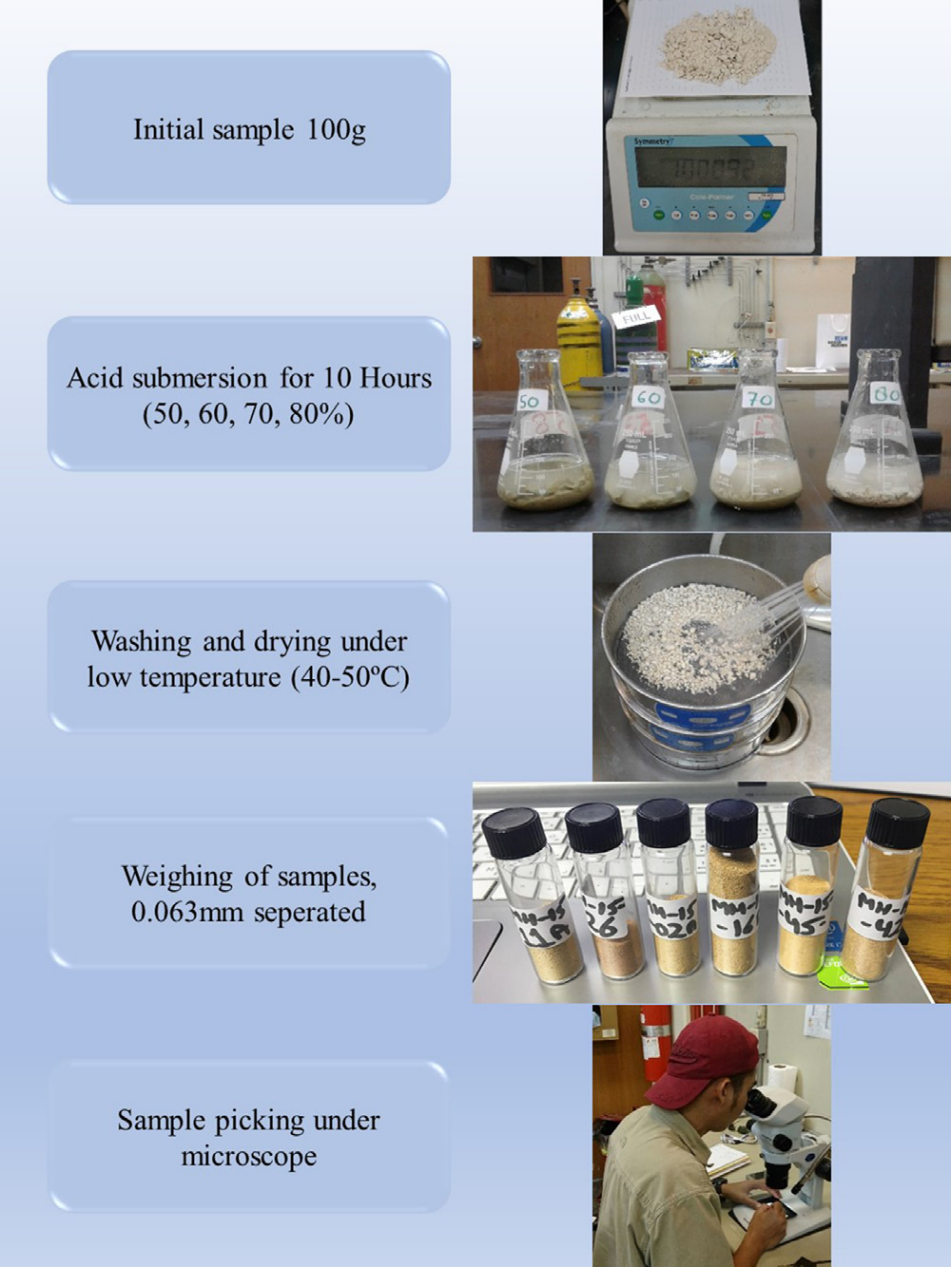
Summary flow chart of the main stages in the sample processing using acetic acid.

**Fig. 4:**
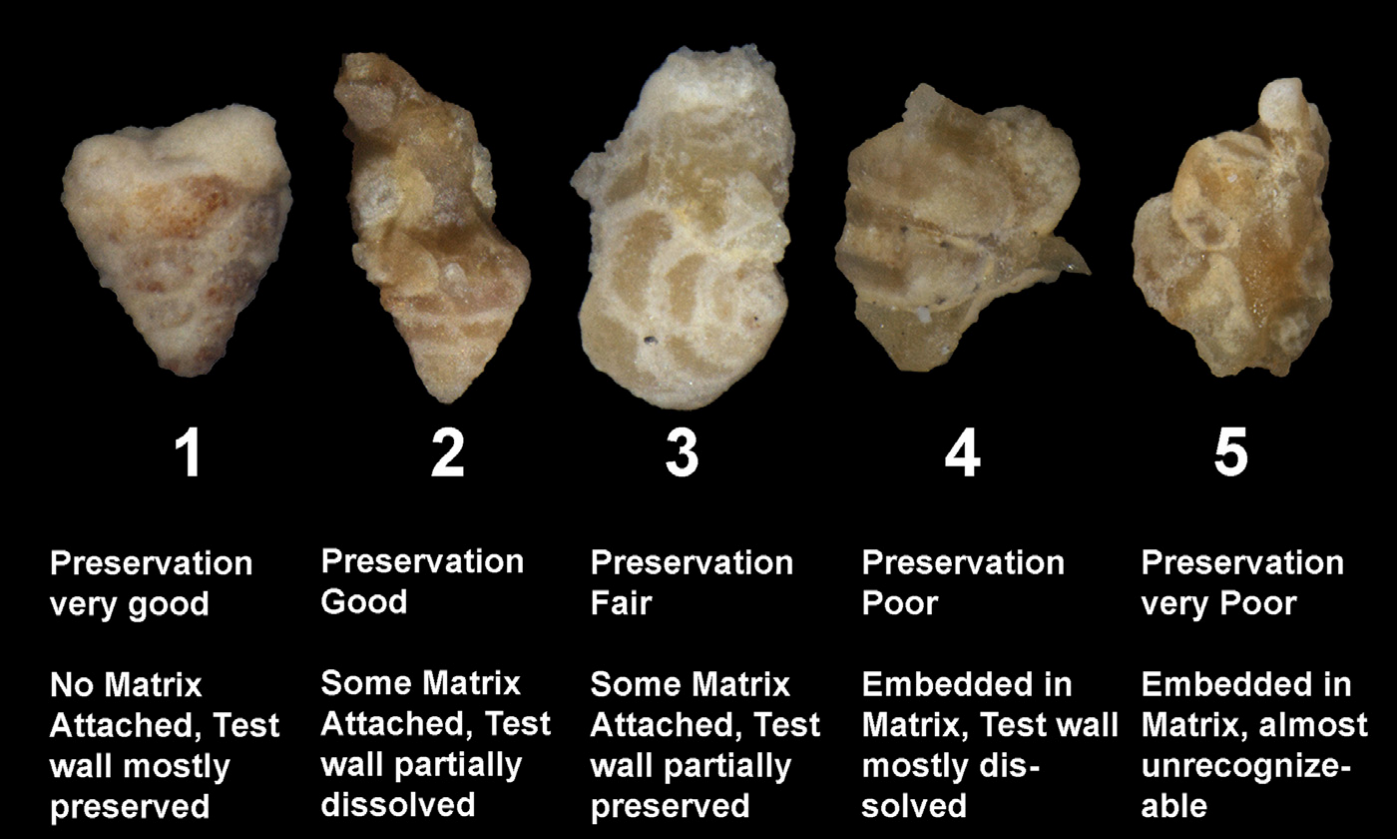
Different preservation states of the foraminifera with preservation ranked from one to five, one is given to clean foraminifera with well-preserved walls while five is assigned to foraminifera covered in matrix with poor wall preservation.
(9) Representative specimens were photographed using a Nikkon-1500 camera microscope.

Polished thin sections were studied at the outset of our study to assess the abundance of microfossils present in the samples. Samples rich in microfossils, especially foraminifera, were selected as potential candidates for acetic acid processing. The samples were subsequently treated with acetic acid using the following steps given below (Fig. 3):

(1) 100 g of carbonate sample was broken down into small fragments of 2–5 mm. The small fragment size is recommended as acid reacts readily on increased surface area and will give better results. However, during crushing of the samples, care should be taken to ensure that the microfossils are not destroyed.
(2) Crushed samples are then placed in glass beakers and are properly labeled.
(3) Solutions of 100%, 90%, 80%, 70%, 60%, and 50% acetic acid (CH3COOH) mixed with 10%, 20%, 30%, 40%, or 50% distilled water, respectively, were used to disaggregate the samples (the level of the acetic acid / water mixture is kept at least 2 cm above the sample level).
(4) The submersed samples with concentrations ranging from 50 to 80% were left in the solution overnight, for at least 10 to 15 h, to help the disaggregation process. For the highest concentration of acid, samples were left for 5 h at 90% and 2 h at 100% concentrations.
(5) The disaggregated samples were wet sieved through stainless steel standard sieves with mesh openings of 1.00, 0.50, and 0.063 mm.
(6) The residue from the 0.063 mm sieve was dried at low temperature (40–50 °C) on a hot plate until completely dry.
(7) The sample residues were transferred to labeled small sample vials. The foraminiferal specimens in the residues were sorted using a binocular stereo microscope. The recovery was assessed by weighing the ˃ 63 µm residue and 300 specimens that were picked from each sample.
(8) The quality of the sample residue was then assessed by determining the preservation state of the recovered specimens. Both dissolved and partially- or undissolved specimens (specimens that still had matrix attached) were picked, counted, and ranked one to five with one being well-preserved and five showing very poor preservation (Fig. 4).

## Results

### Dhruma Formation

**Fig. 5:**
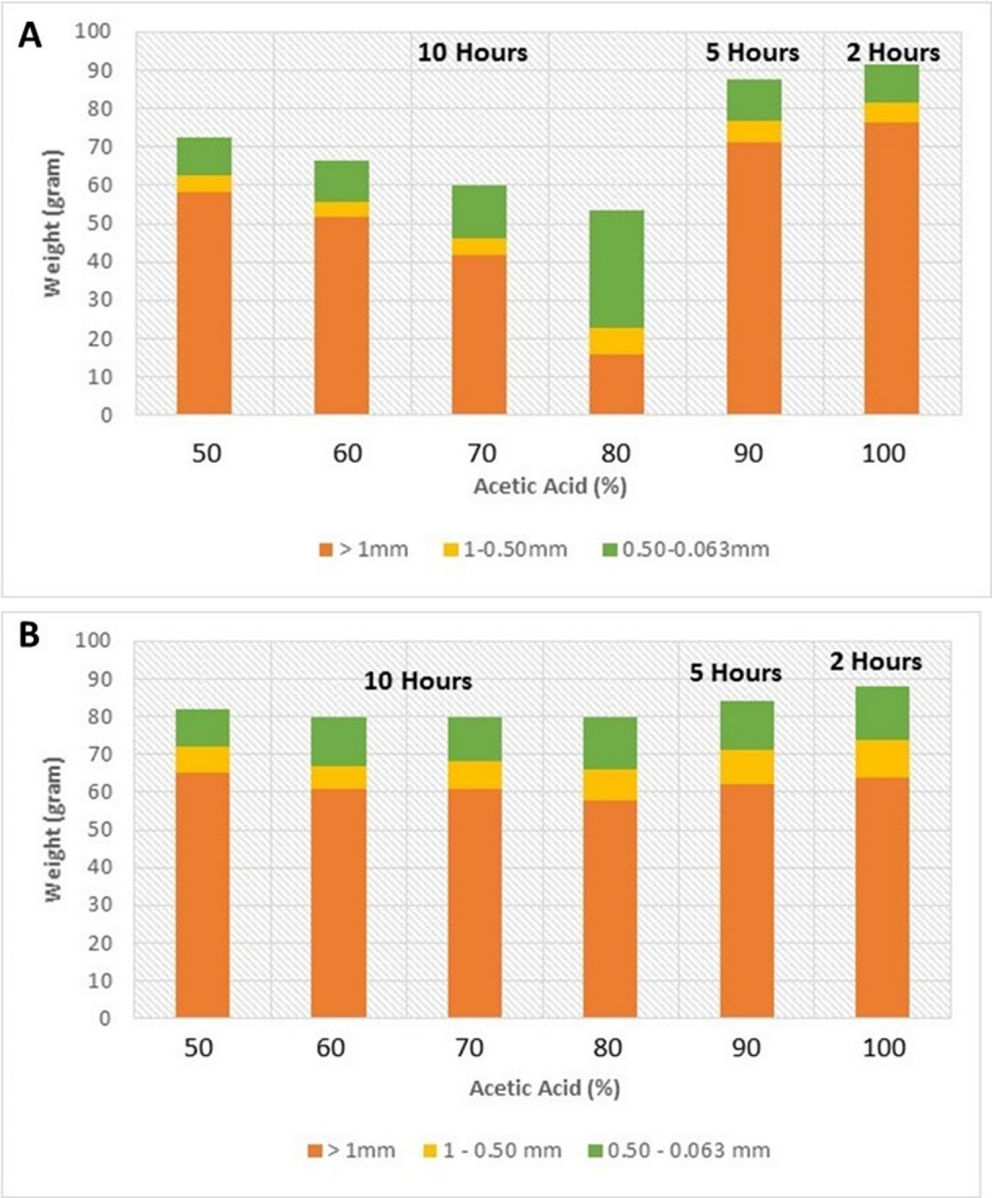
Recovery of acid residues from an initial sample size of 100 g, and proportions of three particle size fractions for different concentrations of acid and reaction times. (A) Jurassic limestones (B) Miocene limestones.

The Dhruma Formation samples show different residue recoveries from different acid concentrations. For acid concentrations of 50, 60, 70 and 80%, which were left to react for 10–15 h, we observed a steady decrease in the weight of the ˃ 63 µm residue as the concentration of acid was increased. Overall, a weight loss of 30% to nearly 50% of the total weight of 100 g was observed. Additionally, the weight of the finest fraction (0.063 mm) increased as a function of acid concentration, which means that the higher concentrations of acid were more effective in disaggregating limestone into finer particles. However, the weight recovery was different for the 90 and 100% concentrations that were left to react for a shorter time. For 90% concentration left for 5 h, we observed better dissolution of samples. This sample has lower weight of higher size residues than the sample that was treated with 100% acid for 2 h only. For the 2 h sample, the weight loss was only 9 to 13% of the initial weight of 100 g, suggesting that the acid needs more time to react (Fig. 5A).

**Fig. 6:**
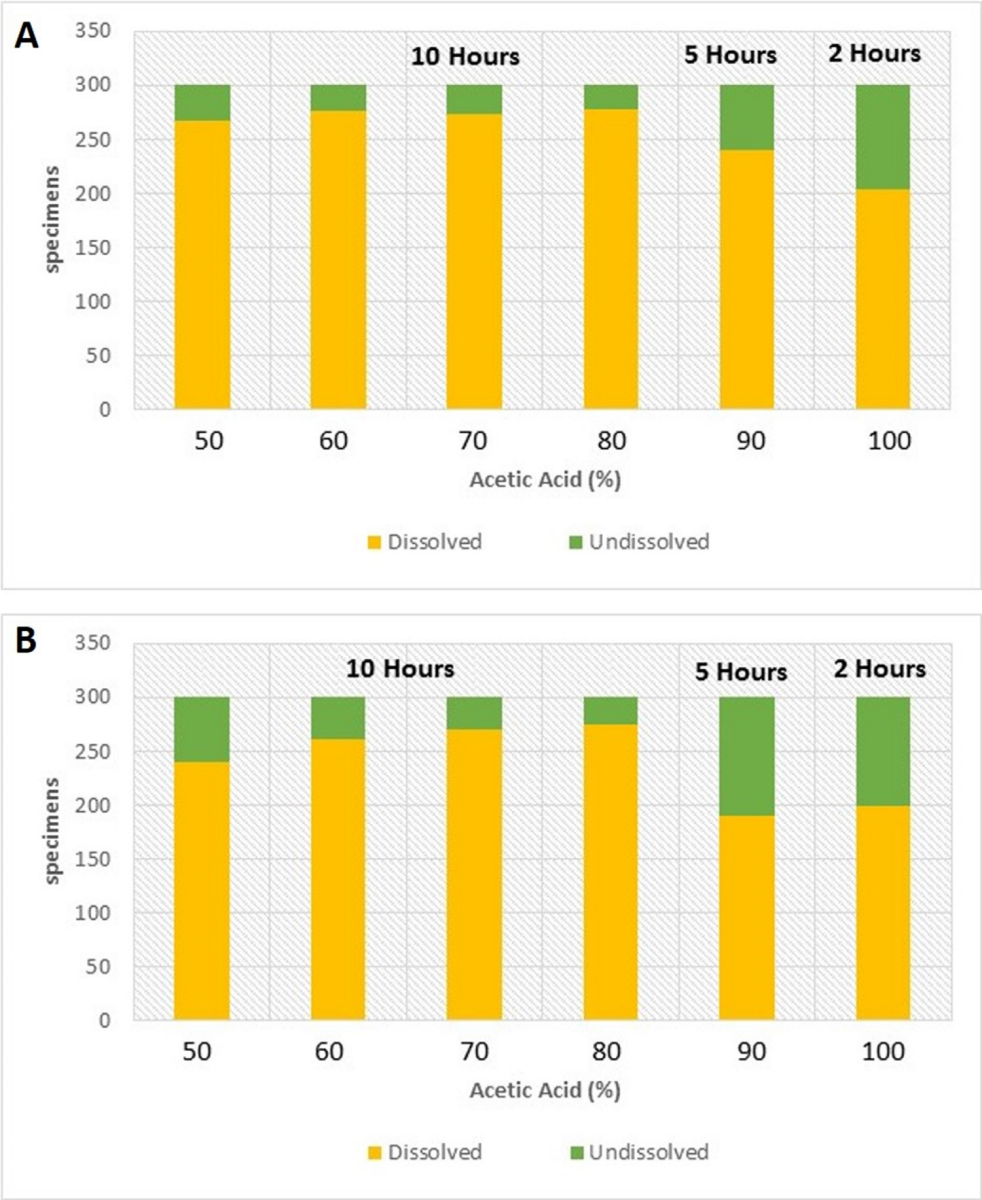
Preservation state of foraminifera in a sample of 300 specimens picked from 3 g of the acid residue from the 0.50–0.063 mm size fraction**.** In general, all concentrations show good recovery of foraminifera from both the Jurassic Dhruma Formation (A) and the Miocene Dam Formation (B).

**Fig. 7:**
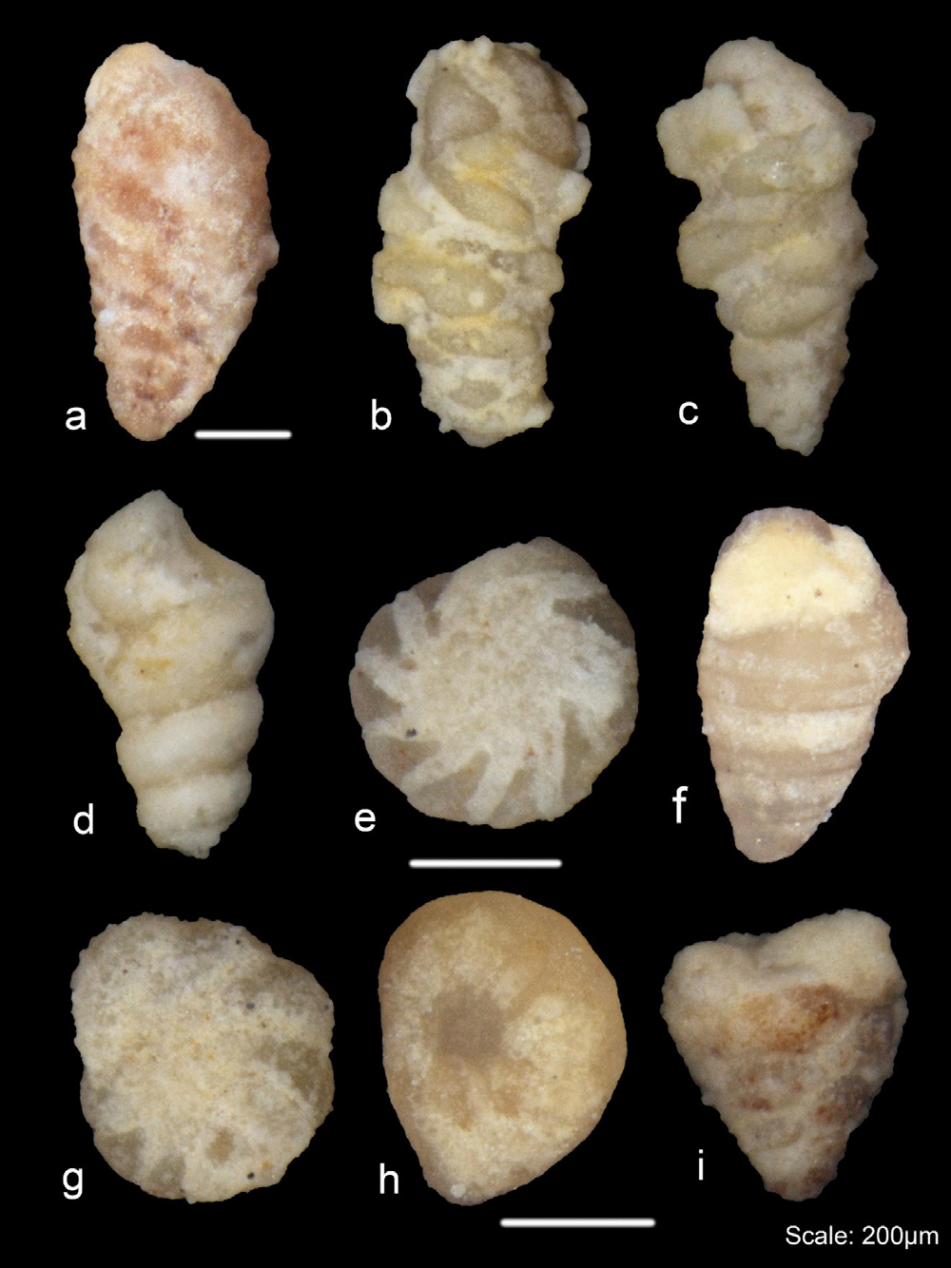
Examples of well-preserved foraminifera recovered from the Dhruma Formation using different concentrations of acetic acid.

**Fig. 8:**
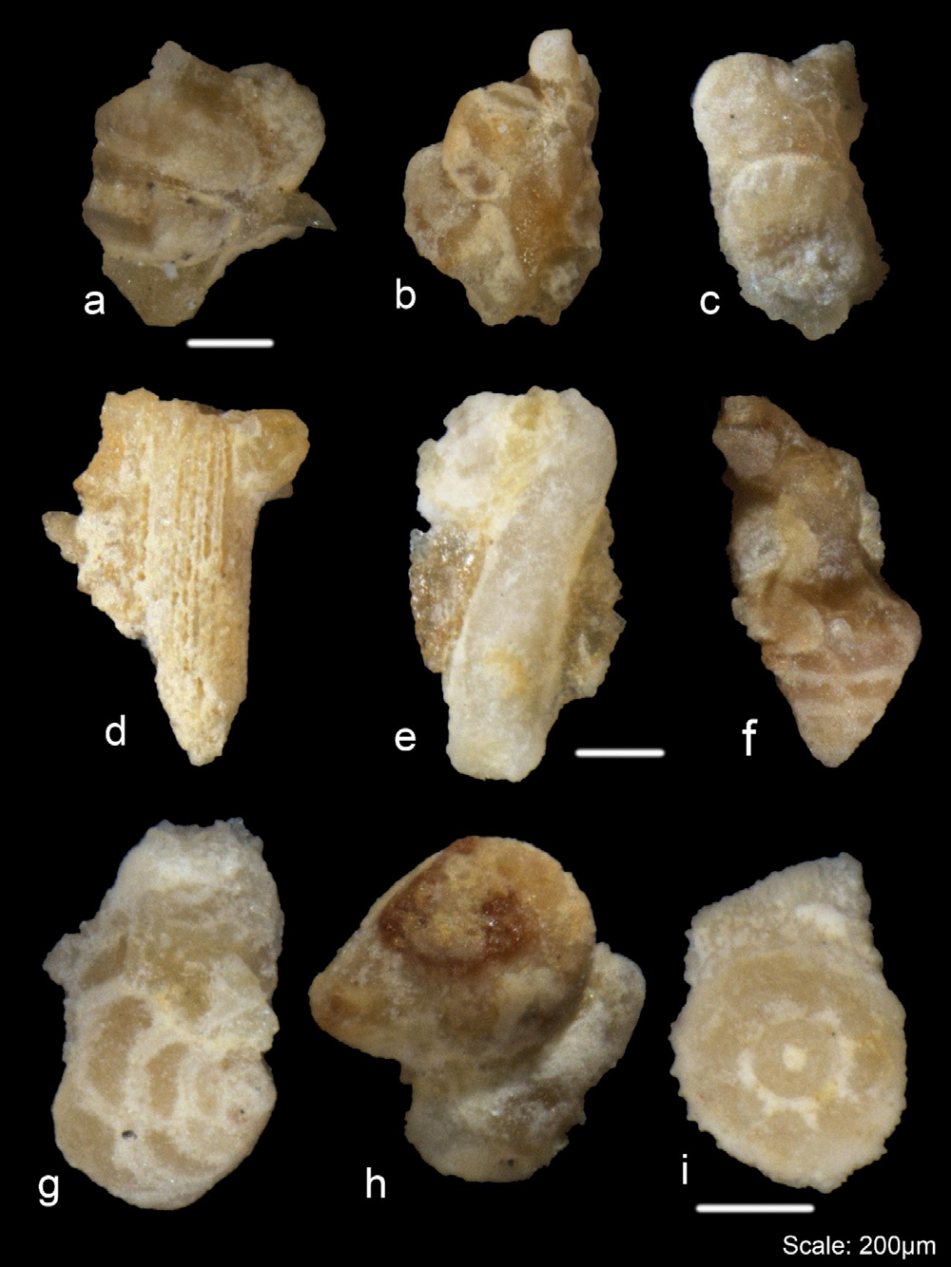
Examples of undissolved foraminifera from the Dhruma Formation with some rock fabric still attached.

In terms of fossil recovery, 300 specimens were picked and examined from each residue recovered from the different acid concentrations. The results from the concentrations from 50 to 80% show the expected trend of high recovery of dissolved specimens from higher concentrations. Some minor differences which include a better recovery of properly dissolved microfossils from the 60% concentration than from the 70% were, however, observed. This can be due to human error (accidental change in acid concentration or different time for reaction). The fossil recovery from the 90% concentration left for 5 h was, however, encouraging when compared with the 100% concentration left for 2 h (Fig. 6A). This also coincides with our previous results from the weight percentages, and we therefore conclude that acid reaction processing time is a crucial component of the acid leaching process. Examples of both dissolved and undissolved specimens from the Jurassic Dhruma Formation are given in Figs. 7 and 8. The different species recovered from each acid concentration are presented in Fig. 11A.

### Miocene Dam Formation

Recovery of acid residues from the Dam Formation samples and granulometric analysis for different concentrations of acid and processing times show that there are slight differences between each concentration and processing time. From an initial sample weight of 100 g, the acetic acid method reduced the weight of the obtained residue by about 18–20 g for 50–80% concentration and 13–14 g for 90 and 100% concentration, with the larger fragments (> 1 mm) accounting for 60–65 g (Fig. 5B).

**Fig. 9:**
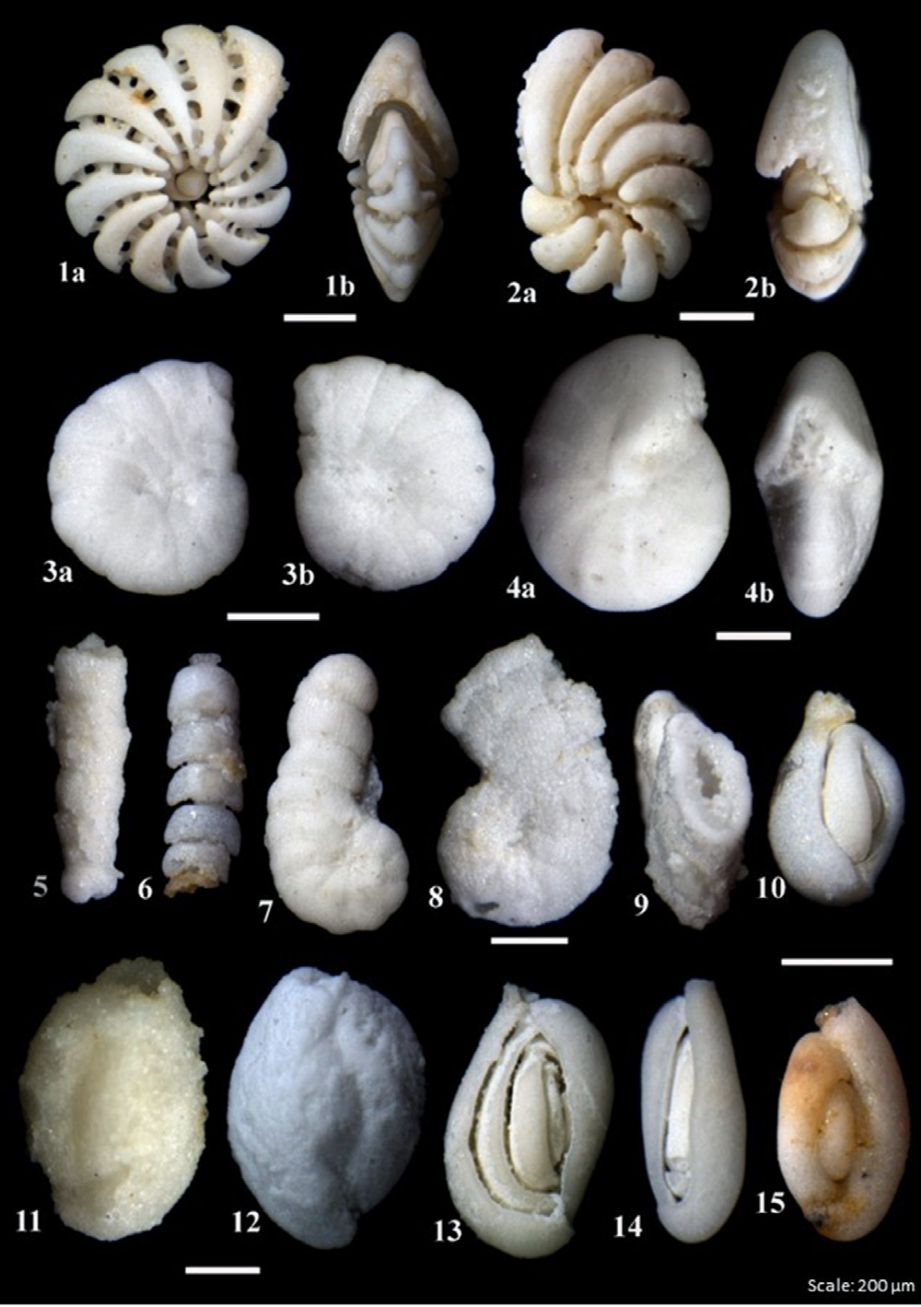
Examples of well-preserved foraminifera from the Dam Formation, showing clean wall surfaces.

**Fig. 10:**
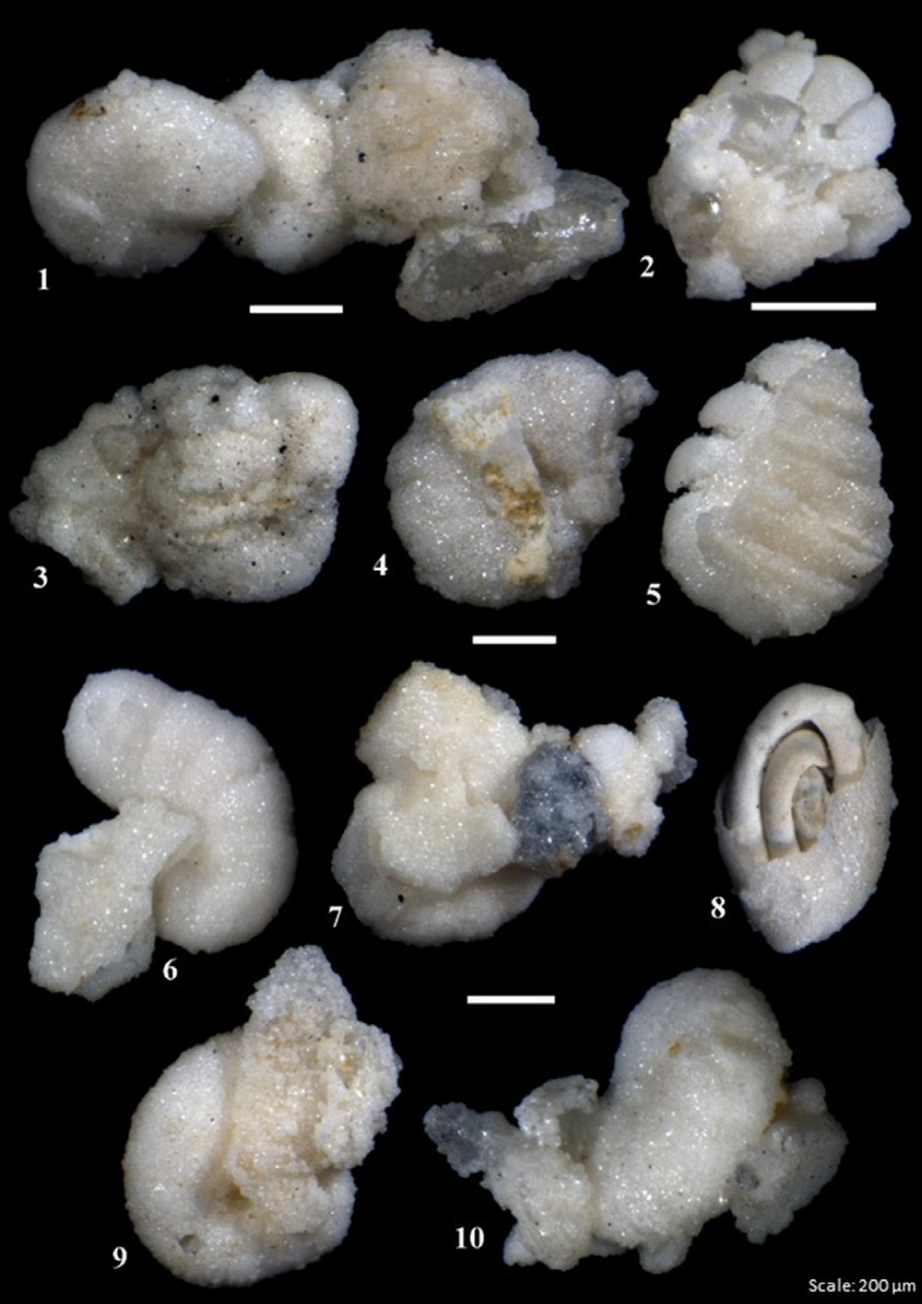
Example of undissolved or partially dissolved foraminifera from the Dam Formation, showing some rock fabric not completely separated from the foraminifers.

**Fig. 11:**
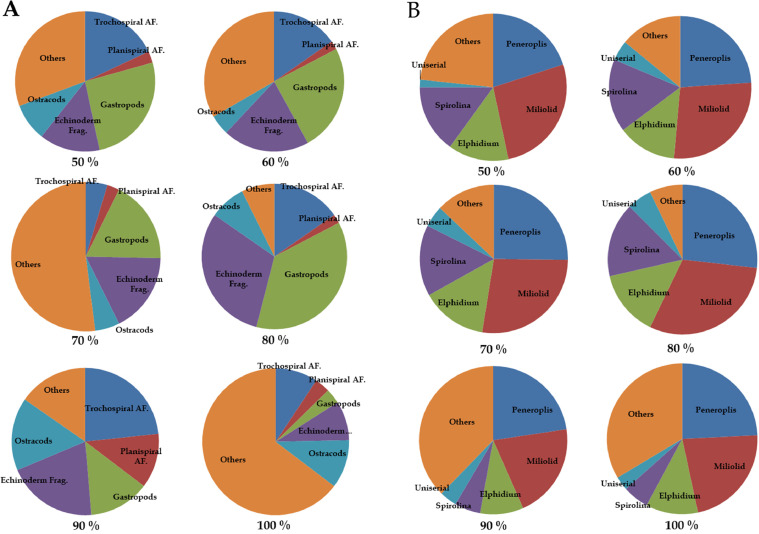
Pie-charts showing different concentrations of several species identified. (A) Shows the microfossil species recovered from Middle Jurassic Dhruma Formation. (B) Shows the species recovered from Middle Miocene Dam Formation. “Others” is used for unrecognized and partially or undissolved microfossils.

The small fraction of residues between 0.50 and 0.063 mm was split into 3 g subfractions and picked to study the diversity and preservation state of foraminifera from different concentrations of acid. The main differences were observed in the proportions of fully dissolved and partially- or undissolved foraminifera present in each concentration. The amount of partially- or undissolved foraminifera decreased from lower concentrations (50%) of acid to high concentrations of acid (80%) for the same processing time (Fig. 6B). On the other hand, for 90% (5 h) and 100% (1 h) a high amount of partially- or undissolved foraminifera were recorded. This is considered as moderate recovery of foraminifera. Examples of dissolved and partially or undissolved foraminifera are shown in Figs. 9 and 10. The different species of microfossils recovered from each acid concentration are shown in Fig. 11B.

The combined summary of our results for both Middle Jurassic Dhruma Formation and the Middle Miocene Dam Formation are summarized in Table 2.

**Table 2:** A summary of results from our study with reaction times, resultant weight fractions and general preservation status from each sample.

Sr #	Sample%	Trochospiral Agglutinated Foraminifera	Planispiral Agglutinated Foraminifera
1	50	2.46	3.50
2	60	3.45	3.66
3	70	3.22	2.50
4	80	3.0	2.66
5	90	3.47	2.66
6	100	3.66	3.66

## Discussion

In our experiment we noted that the effectiveness of the acid reaction differs between lithologies. This may be due to the differences in the carbonate facies and matrix composition. For example, compared to the Jurassic samples, which are predominantly composed of skeletal and non-skeletal grains and a lesser amount of matrix, the Miocene samples have high matrix contents (Figs. 2 and 3). In the results, this difference is observed in terms of weight percentages recovered. We recorded a greater weight loss in Jurassic samples compared to the Miocene ones (Fig. 5). The main reason might be due to the fact that the acid dissolved most of the matrix in Jurassic samples quickly, but because the Miocene samples were matrix dominated, the acid was not able to dissolve all of it. This finding is supported by the results of Tarsilli and Warne [40], who showed that approximately 20–30 g of undissolved samples remaining were grain dominated, whereas 70–90 g of undissolved samples remaining had a high percentage of micrite and sparry calcite present as matrix.

Over 300 specimens were picked from residues obtained from different concentrations of acid processing for both Dhruma and Dam Formation samples. The main difference for each concentration was the quality and amount of fully dissolved and partially or undissolved foraminifera: either the microfossil is still attached to the matrix, or it is completely removed and has a cleaner surface (Fig. 4). In general, the samples soaked in 60–80% acid concentrations over 10 h processing time (Fig. 6) are categorized as having good recovery. At 90 and 100% concentrations, recovery was categorized as fair to moderate. In both concentrations, at least 100 out of the 300 specimens counted were found to still be attached to the rock matrix (Fig. 6). Although some of these specimens were found attached to grains, they were recognizable and were identified at least to the generic level while the cleaner specimens were easily identified to species level with the help of images taken in dorsal, ventral, and umbilical views.

A detailed study of these acid percentages by the authors of the middle D5-D6 units of the Dhruma Formation shows promising results [39]. The diversity of foraminifera species recovered was far higher than any previous study done on these units. From the D5-D6 units, 35 foraminiferal species belonging to 19 different genera were extracted. Some species, including *Everticyclammina praevirguliana, Nautiloculina oolithica, Redmondoides lugeoni, Siphovalvulina variabilis, Siphovalvulina colomi,* and *Pseudomarssonella maxima,* were identified which were never observed from these units with the use of polished thin sections. Additionally, five species of a single genus, *Redmondoides,* were identified for the first time. Using these data, stratigraphic ranges for some foraminiferal species were established in the studied units while some previous ranges were extended.

For the Miocene Dam Formation samples, it was difficult to check the shell surface quality as a result of acetic acid treatment for different concentrations because most of the microfossil specimens in the samples occur as molds, with their outer walls dissolved during diagenesis (Fig. 8). This may be due to the presence of mostly calcareous porcelaneous species (almost 75%) along with some hyaline genera (24%) with only a minor percentage of agglutinated foraminifera (1%) in the formation. However, signs of acid corrosion are evident in the Jurassic Dhruma samples as well. When the concentration of acid was increased, we see a marked evidence of acid action on the fossil surfaces. The recovered microfossils, especially the agglutinated foraminifera, were studied for increased dissolution of the wall texture at different acid concentrations and were assigned a rank value from 1 to 5. A rank value of 5 was given to the fossils with complete loss of wall material, resulting in preservation of only internal molds, while a value of 1 was given to the well-preserved microfossils (Fig. 4). With an increasing concentration of acid, we see a decrease in wall preservation, which may be due to the acid action on the foraminiferal test wall. Most of the fossils from different concentrations were moderately preserved, and rankings between 2 and 4 were assigned. Overall small differences in the average rank values can be observed between the difference acid concentrations (Table 3). A detailed study on four outcrops of the Miocene Dam Formation was also done by the authors using the similar technique [38]. A total of 46 species belonging to 24 genera and 16 families were recovered. Morphotypes and morphogroups of the extracted microfossils were determined with the help of detailed test morphology including chamber arrangement.

**Table 3:** Ranking of wall texture and preservation of foraminifera from Middle Jurassic Dhruma Formation. (5= Wall not preserved at all and only infillings recovered, or foraminifera completely covered in matrix, 4= Wall preserved at sutures but chamber wall often not preserved or foraminifera mostly covered in matrix, 3= Some chambers showing infillings or foraminifera partially covered in matrix, 2= Wall is mostly preserved intact or only minor amounts of matrix attached with foraminifera, 1= Very good wall preservation or clean foraminifera).

Acid Concentration (%)	Initial Sample size (mm)	Reaction Time (hours)	Total Weight of Residue (gram)		General Preservation status
			Jurassic Dhruma Fm	Miocene Dam Fm	
50	2–5	10	75	83	(3) Medium
60	2–5	10	66	81	(4) Good
70	2–5	10	59	81	(4) Good
80	2–5	10	53	80	(5) Very Good
90	2–5	5	87	86	(3) Medium
100	2–5	2	91	87	(1) Poor

The initial particle size of the crushed sample can also influence the results, as acid reacts more quickly with smaller fragments. Therefore, small-sized homogeneous samples are recommended to substantially increase the surface area for reaction. Care should be taken while crushing the samples to preserve the microfossil content, and therefore fine crushing should be avoided.

Therefore, different concentrations of acetic acid can be used for different purposes. If the study requires normal recovery and there is no time constraint, we recommend using a lower concentration of acid (60%), which is more environmentally friendly and was found to be the optimum concentration needed in our study. However, for better results, acid concentrations can be increased as desired. For routine micropaleontological research, acetic acid concentrations from 60 to 80% with reaction times of 10–15 h can be used. If quick results are required in an industrial setting in order to check the fossil content and quantify biogenic proportions, such as during drilling monitoring and bio-steering [41], we recommend the highest concentrations with a shorter reaction time, as a moderate microfossil recovery can still be obtained.

## Conclusions

We conclude that nearly all concentrations of acetic acid tested yielded promising results for samples of Jurassic and Miocene age. The best recovery of microfossils was observed for higher concentrations of acid left for a longer reaction time.

In terms of weight percentages of the obtained residue, we obtained similar trends for both Jurassic and Miocene limestones. For acid concentrations between 50 and 80%, the weight percentage of particle size > 1 mm shows an inverse relationship to acid concentration. Similarly, the weight percent of the smallest size particles above 0.063 mm increases with higher acid concentrations.

The 90% acid concentration left for 5 h shows better results compared with the 100% concentration left for two hours and samples processed with 90 and 100% acetic acid left for shorter reaction times show lower recovery compared with samples processed at lower concentrations with longer reaction times. The acid reacts best when a more concentrated solution is used for a longer time. This also suggests that a minimum time for the reaction to take place is a crucial factor for the method to be effective. A concentration of 60% acid is suggested to be the optimal concentration for routine micropaleontological work in the lithified carbonate rocks of the Middle East.

## Ethics statements

*If your work involved human subjects****:*** No, our work does not involve any human subjects.
*If your work involved animal experiments****:*** No, our work does not involve any animal experiments.
*If your work involved data collected from social media platform****:*** No, no data from social media platforms was utilized in this research.

## Supplementary material and/or additional information

Not applicable.

## CRediT authorship contribution statement

**Muhammad Hammad Malik:** Investigation, Resources, Formal analysis, Data curation, Writing – original draft, Writing – review & editing. **Septriandi A. Chan:** Investigation, Resources, Formal analysis, Data curation, Writing – original draft, Writing – review & editing. **Lamidi O. Babalola:** Supervision, Resources, Writing – review & editing. **Michael A. Kaminski:** Conceptualization, Funding acquisition, Project administration, Writing – review & editing.

## Declaration of Competing Interest

The authors declare that they have no known competing financial interests or personal relationships that could have appeared to influence the work reported in this paper.
